# Protective effects of carbonic anhydrase inhibition in brain ischaemia *in vitro* and *in vivo* models

**DOI:** 10.1080/14756366.2021.1907575

**Published:** 2021-05-31

**Authors:** Ilaria Dettori, Irene Fusco, Irene Bulli, Lisa Gaviano, Elisabetta Coppi, Federica Cherchi, Martina Venturini, Lorenzo Di Cesare Mannelli, Carla Ghelardini, Alessio Nocentini, Claudiu T. Supuran, Anna Maria Pugliese, Felicita Pedata

**Affiliations:** a Department of Neuroscience, Psychology, Drug Research and Child Health (NEUROFARBA), Division of Pharmacology and Toxicology, University of Florence, Florence, Italy; b Department of Neuroscience, Psychology, Drug Research and Child Health (NEUROFARBA), Section of Pharmaceutical Sciences, University of Florence, Florence, Italy

**Keywords:** Carbonic anhydrase inhibitors, synaptic potentials, anoxic depolarisation, cerebral ischaemia, middle cerebral artery occlusion

## Abstract

Ischaemic stroke is a leading cause of death and disability. One of the major pathogenic mechanisms after ischaemia includes the switch to the glycolytic pathway, leading to tissue acidification. Carbonic anhydrase (CA) contributes to pH regulation. A new generation of CA inhibitors, AN11-740 and AN6-277 and the reference compound acetazolamide (ACTZ) were investigated in two models of brain ischaemia: in rat hippocampal acute slices exposed to severe oxygen, glucose deprivation (OGD) and in an *in vivo* model of focal cerebral ischaemia induced by permanent occlusion of the middle cerebral artery (pMCAo) in the rat. *In vitro*, the application of selective CAIs significantly delayed the appearance of anoxic depolarisation induced by OGD. *In vivo*, sub-chronic systemic treatment with AN11-740 and ACTZ significantly reduced the neurological deficit and decreased the infarct volume after pMCAo. CAIs counteracted neuronal loss, reduced microglia activation and partially counteracted astrocytes degeneration inducing protection from functional and tissue damage.

## 1. Introduction

Hypoxic–ischaemic insult to the brain generally causes necrosis, although in most cases there exists also a mechanism of delayed and apoptotic type damage in the region (penumbra) surrounding the area of most severe injury (core)^1–4^. In the adult mammalian central nervous system (CNS), neurons injured by ischaemia normally have only limited ability or fail to regenerate axons, resulting in long lasting disabilities in sensory, motor, or cognitive functions^5^. To date stroke is considered the second most common cause of death and a major cause of long-term disability worldwide. Ischaemic stroke is caused from occlusion of a major cerebral artery by a thrombus or an embolism and it commonly accounts for approximately 80% of all stroke cases. The occlusion leads to a reduction of cerebral blood flow rate, a condition of hypoxia and glucose deprivation (oxygen, glucose deprivation: OGD) and subsequent tissue damage in the affected region^6^. Hypoxia/ischaemia triggers a complex sequence of pathophysiological events that evolve over time which results in brain damage. First, primary acute mechanisms consist in impairment of the oxidative phosphorylation of glucose due to the low levels of oxygen present, thus most energy derives from the alternative, anaerobic glycolytic pathway, which leads to lactic acid formation and consequent ambient acidification^7^^,^^8^. Excitotoxicity and peri-infarct depolarizations are followed by a chronic secondary damage caused by the activation of resident immune cells, i.e. microglia, and production or activation of inflammation mediators^9^^,^^10^. In the last years basic research produced several protective pharmacologic agents in animal models of brain ischaemia. Unfortunately, during clinical trials drugs resulted to be inefficacious and the only successful pharmacological treatment approved to date is tissue plasminogen activator that brings to decrease ischaemia associated thrombosis risk^11^. However, because of its narrow therapeutic time-window, thrombolytic application is very limited in clinical applications^12^.

Carbonic Anhydrase (CA, EC 4.2.1.1) is the enzyme responsible for converting carbon dioxide into a hydrogen ion and a bicarbonate ion, thus contributing to multiple biological processes, including pH regulation^13–15^. In hypoxic/anoxic conditions, *in vitro* studies document a decrease in pH in neurons and glial cells^16^. The evidence that under hypoxic conditions, two CA isoforms, IX and XII, increase through the hypoxia inducible factor^17–20^ allows to hypothesise a possible CA relevance in ischaemia. CA inhibitors (CAIs) could therefore contribute to pH homeostasis under brain ischaemia by reducing the concentration of hydrogen ions^13^^,^^21–24^. On the other hand, CA inhibition has a lot of effects not only related to the diuretic action and potentially useful also in brain pathologies^13^^,^^24^.

**Figure 1.**
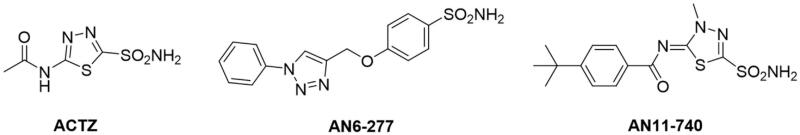
Chemical structure of the investigated CAIs: acetazolamide (ACTZ) and newly reported sulphonamide CAIs.

The purpose of this study was to investigate the protective effect of new generation sulphonamide CAIs such as AN11-740 and AN6-277 (Figure 1)^25^^,^^26^, in two models of brain ischaemia: an *in vitro* model of rat hippocampal acute slices that underwent severe, 30 min long OGD episodes^27^ and an *in vivo* model of focal cerebral ischaemia induced by permanent occlusion of the middle cerebral artery (pMCAo) in the rat^28^.

## 2. Materials and methods

### 2.1. Carbonic anhydrase inhibition

An Applied Photophysics stopped-flow instrument has been used for assaying the CA catalysed CO_2_ hydration activity^29^. Phenol red (at a concentration of 0.2 mM) has been used as indicator, working at the absorbance maximum of 557 nm, with 20 mM Hepes (pH 7.5) as buffer and 20 mM Na_2_SO_4_ (for maintaining constant the ionic strength), following the initial rates of the CA-catalysed CO_2_ hydration reaction for a period of 10 − 100 s. The CO_2_ concentrations ranged from 1.7 to 17 mM for the determination of the kinetic parameters and inhibition constants. For each inhibitor, at least six traces of the initial 5 − 10% of the reaction have been used for determining the initial velocity. The uncatalyzed rates were determined in the same manner and subtracted from the total observed rates. Stock solutions of inhibitor (0.1 mM) were prepared in distilled − deionized water and dilutions up to 0.01 nM were done thereafter with the assay buffer. Inhibitor and enzyme solutions were preincubated together for 15 min at room temperature prior to assay in order to allow for the formation of the E − I complex. The inhibition constants were obtained by nonlinear least-squares methods using PRISM 3 and the Cheng − Prusoff equation and represent the mean from at least three different determinations. CA isoforms were recombinant ones obtained in-house as reported earlier and their concentrations in the assay system ranged between 3 and 10 nM^25^. AN11-740 and AN6-277 and the reference compound acetazolamide (ACTZ) produce an effective inhibition of the hypoxia-associated CA IX and XII and CA I and II, the most physiologically relevant isoforms (Table 1). Moreover, the highly lipophilic character of these CAIs^26^ favours permeability through the Blood Brain Barrier (BBB).

**Table 1.** Inhibition data of CA isoforms I, II, IX, and XII by inhibitors ACTZ, AN6-277, and AN11-740 by a stopped flow CO_2_ hydrase assay^29^.

Cmpd	KI (nM)^a^			
	CA I	CA II	CA IX	CA XII
ACTZ	250 ± 15	12 ± 0.8	25 ± 1.4	5.7 ± 0.5
AN6-277^b^	565.6 ± 34.2	1.2 ± 0.1	2.6 ± 0.2	1.1 ± 0.1
AN11-740	1580 ± 110^c^	5.9 ± 0.4 ^c^	1.3 ± 0.1	3.0 ± 0.2

^a^Inhibition data are expressed as means ± SEM of 3 different assays; ^b^data from ref^25^; ^c^data from ref^26^.

### 2.2. *In vitro* experiments

All animal experiments were performed according to the Italian Law on Animal Welfare (DL 26/2014), approved by the Institutional Animal Care and Use Committee of the University of Florence and by the Italian Ministry of Health. All efforts were made to minimise animal sufferings and to use only the number of animals necessary to produce reliable scientific data. Male Wistar rats (Envigo, Italy, 150–200 g body weight) were used. Experiments were carried out on rat hippocampal acute slices, prepared as previously described^30^^,^^31^.

#### 2.2.1. Preparation of slices

Animals were killed with a guillotine under anaesthesia with isoflurane (Baxter, Rome, Italy) and hippocampi were rapidly removed and placed in ice-cold oxygenated (95% O_2_–5% CO_2_) artificial cerebrospinal fluid (aCSF) of the following composition (mM): NaCl 125, KCl 3, NaH_2_PO_4_ 1.25, MgSO_4_ 1, CaCl_2_ 2, NaHCO_3_ 25, and D-glucose 10. Slices (400 μm nominal thickness) were cut using a McIlwain Tissue Chopper (Mickle Laboratory Engineering Co. Ltd., Gomshall, United Kingdom) and kept in oxygenated aCSF for at least 1 h at room temperature. A single slice was then placed on a nylon mesh, completely submerged in a small chamber (0.8 ml) and superfused with oxygenated aCSF (31–32 °C) at a constant flow rate of 1.5 ml/min. The treated solutions reached the preparation in 60 s and this delay was considered in our calculations.

#### 2.2.2. Extracellular recordings

Test pulses (80 μs, 0.066 Hz) were delivered through a bipolar nichrome electrode positioned in the stratum radiatum of the CA1 region of the hippocampus to stimulate the Schaffer collateral-commissural pathway. Evoked potentials were extracellularly recorded with glass microelectrodes (2–10 MΩ, Crisel Instruments for Harvard Apparatus LTD, United Kingdom) filled with 150 mM NaCl. The recording electrode was placed at the dendritic level of the CA1 region to record field excitatory postsynaptic potentials (fEPSPs). Responses were amplified (200×, BM 622, Mangoni, Pisa, Italy), digitised (sample rate, 33.33 kHz), and stored for later analysis with LTP (version 2.30 D) program^32^. The amplitude of fEPSP was always measured as the difference between the negative peak following the afferent fibre volley and the baseline value preceding the stimulus artefact. When a stable baseline of evoked responses was reached, fEPSP amplitudes were routinely measured and expressed as the percentage of the mean value recorded 5 min before the application of any treatment (in particular pre-OGD, not shown). Stimulus-response curves were obtained by gradual increase in stimulus strength at the beginning of each experiment. The test stimulus strength was then adjusted to produce a response whose amplitude was 40% of the maximum and was kept constant throughout the experiment. Simultaneously, with fEPSP amplitude, anoxic depolarisation (AD) was recorded as negative extracellular direct current (d.c.) shifts induced by OGD. The d.c. potential is an extracellular recording considered to provide an index of the polarisation of cells surrounding the tip of the glass electrode^33^. AD latency, expressed in min, was calculated from the beginning of OGD; AD amplitude, expressed in mV, was calculated at the maximal negativity peak. In the text and bar graphs, AD amplitude values were expressed as positive values.

#### 2.2.3. Application of OGD and drugs

OGD was obtained by superfusing the slice with aCSF without glucose and gassed with nitrogen (95% N_2_ −5% CO_2_)^34^. This caused a drop in *p*O_2_ in the recording chamber from ∼500 mm Hg (normoxia) to a range of 35–75 mm Hg (after 7-min OGD)^35^. Throughout the manuscript, the terms “untreated OGD slices” or “treated OGD slices” refer to hippocampal slices in which OGD episodes of different duration were applied in the absence or in the presence of drugs, respectively. ACTZ and the CAIs, AN11-740 and AN6-277, were applied 20 min before and during OGD application. All drugs were dissolved in dimethyl sulphoxide (DMSO). Stock solutions, of 1000–10,000 times the desired final concentration, were stored at −20 °C. The final concentration of DMSO (0.05 and 0.1% in aCSF) used in our experiments did not affect either fEPSP amplitude or the depression of synaptic potentials induced by OGD (data not shown).

#### 2.2.3. Statistical analysis

Statistical significance was evaluated by Student’s paired or unpaired t-tests. Analysis of variance (one-way ANOVA), followed by Bonferroni multiple comparison *post hoc* test was used, as appropriate. *p* Values from both Student’s paired and unpaired *t*-tests are two-tailed. Data were analysed using software package GraphPad Prism (version 7.0; GraphPad Software, San Diego, CA, United States). All numerical data are expressed as the mean ± standard error of the mean (SEM). A value of *p* < 0.05 was considered significant.

#### 2.3. *In vivo* experiments

##### 2.3.1. Animals

Male Wistar rats (Envigo, Italy) weighting 270–290 g were used. Animals were housed in groups of three with free access to food and water and kept under standardised temperature, humidity and light conditions (12 h light/dark cycle) within the animal house facility of the University of Florence. The experimental procedures were conducted in accordance with the ARRIVE guidelines and were approved by the local Animal Welfare Body (AWB) of the University of Florence and authorised by the Italian Ministry of Health. The ethical policy of the University of Florence complies with to the Directive 2010/63/EU of the European Parliament and to the Italian Regulation DL 26/2014 on the protection of animals used for scientific purposes. According to the law, all efforts were made to fulfil to the principle of 3Rs.

#### 2.3.2. Surgery

Focal cerebral ischaemia was induced permanently by intraluminal MCAo in the right hemisphere. The animals were anaesthetised with 5.0% isoflurane (Baxter International) and spontaneously inhaled 1.0–2.0% isoflurane in air by the use of a mask. Body core temperature was maintained at 37 °C with a recirculating pad and K module and was monitored via an intrarectal type T thermocouple (Harvard, Kent, UK). The surgical procedure to occlude the MCA consisted in insertion of a 4–0 nylon monofilament (Doccol corporation, USA), via the external carotid artery into the internal carotid artery in order to block the origin of the MCA according to the procedure described by^28^. Carprofen (5 mg/kg) was administered intraperitoneally (i.p.) to reduce post-operative pain. The sham operation was conducted by inserting the filament into the internal carotid artery and immediately withdrawing it.

#### 2.3.3. Drug administration and experimental groups

CAIs 5-acetamido-1,3,4-thiadiazole-2-sulphonamide (ACTZ) and the more lipid soluble compound, AN11-740^26^, were dissolved in saline with 0.05% and 0.01% DMSO, respectively. ACTZ and AN11-740 were sub-chronically administered intraperitoneally (i.p.) at the dose of 4.4 and 1.0 mg/kg, respectively, 5 min, 6 and 20 h after starting MCAo. Doses of CAIs were calculated on the bases of the concentration that revealed protective in OGD experiments (20 µM ACTZ; 3 µM AN11-740).

Animals subjected to pMCAo were sacrificed 24 h after ischaemia. Rats were randomly allocated in the following groups: (1) sham-operated rats (*n* = 7): did not receive any treatment; (2) MCAo + vehicle group (*n* = 4): saline with DMSO administered i.p. 5 min, 6 and 20 h after starting pMCAo; (3) MCAo + ACTZ group (*n* = 4): ACTZ administered i.p. 5 min, 6 and 20 h after starting pMCAo; (4) MCAo + AN11-740 group (*n* = 5): AN11-740 administered i.p. 5 min, 6 and 20 h after starting pMCAo.

#### 2.3.4. Neurological deficit

The neurological deficit was evaluated by the modified Neurological Severity Score (mNSS) test described in ref.^36^. The examiners were blind both to the type of surgery and to the treatment. All tests were carried out before and 24 h after pMCAo. The mNSS test evaluates the sensorimotor deficit: it is composed of motor, sensory, reflex and beam balance tests. The score assigned to each rat at completion of the evaluation equals the sum of all test scores. The test is graded on a scale from 0 (normal score) to 18 (maximal deficit score). In the beam balance test, a score between 0 (normal score) and 6 (maximal deficit score) was assigned to each animal in function of the ability to stay and walk on the beam. Beam balance test score affects 1/3 of the total mNSS score.

#### 2.3.5. Ischaemic brain damage

Rats were anaesthetised with Zoletil 50/50 (100 mg/kg i.p., Virbac, Carros, Francia) and were perfused transcardially with an ice-cold 4% paraformaldehyde solution (in phosphate buffer, pH 7.4). Brains were post-fixed overnight and cryoprotected in a 18% sucrose solution (in phosphate buffer) for at least 48 h. Brains were cut with a cryostat and coronal sections (30 µm) were collected at 210 µm intervals at 12 different levels through the striatum (AP: from +2.2 mm to −1.5 mm from Bregma)^36^. Brain slices were stained by acetate cresyl violet (1%) or by haematoxylin and eosin (H&E). Histological analysis by cresyl violet staining allows to clearly define the infarct area and volume up to 1 week after ischaemia^37^. To evaluate area and volume of ischaemic damage, 12 cresyl violet-stained brain sections per animal were placed directly on the scanning screen of a colour flatbed scanner (CanoScan LiDE 90; Canon). Following image acquisition, the images were analysed using ImageJ software. The measurements of infarct area in striatum and cortex were obtained by manually outlining the margins of infarcted area. Ischaemic cortical and striatal volumes were calculated by multiplying the infarcted area by the slice thickness and summing the volume of the 12 slices. After H&E staining, heterochromatic nuclei were counted at Bregma level within an optical field at 20X in ischaemic cortex and ischaemic striatum. Quantitative analysis was conducted blind to the treatment group and data were then averaged and expressed as mean ± SEM of number cells per optical field of “n” animals.

#### 2.3.6. Neuronal damage and gliosis

Coronal sections (30 µm), stored at −20 °C in antifreeze solution (30% ethylene glycol, 30% glycerol in phosphate buffer) until assay, were mounted on gelatin-coated slides and washed with phosphate buffer saline, 0.3% Triton X-100 (PBS-TX) (for 3 times, 1 min each), blocked with blocking buffer (5 mg/ml of Bovine Serum Albumin/PBS-TX) for 1 h at room temperature. Sections were then incubated overnight at 4 °C with the primary mouse monoclonal antibody antineuronal nuclei (NeuN, specific for neurons, 1:400, Product code: #MAB377; Millipore, Billerica, MA, United States), with mouse monoclonal antibody, anti-Glial Fibrillary Acidic Protein (GFAP, specific for astrocytes, 1:300, Product code: #610565; BD Transduction Laboratories) and with rabbit polyclonal antibody, anti-ionized calcium binding adaptor molecule 1 (IBA1, specific for microglia, 1:300; Product code: #016–20001; Wako Chemicals) dissolved in blocking buffer. The day after, the primary antibody was removed. Slices were washed several times with PBS-TX solution and incubated, in the dark, for 2 h at RT with fluorescent secondary antibodies AlexaFluor 594 donkey anti-mouse (1:400, Product code: #A21203; Invitrogen, Carlsbad, CA, United States) and AlexaFluor 488 goat anti-rabbit (1:400, Product code #A32731; Thermo Fisher Scientific, Altrincham, UK). The sections were mounted onto gelatin-coated slides using Vectashield with DAPI (Vector Laboratories). Slices were observed with an epifluorescent Olympus BX63 microscope (Olympus, Hamburg, Germany) and photographed using a digital camera (Olympus DP50). Images were assembled into montages using Adobe Photoshop Cs 6.1 (Adobe Systems, Mountain view, CA, USA). To quantify neurons, astrocytes and microglia, cells were counted within an optical field at 20X in ischaemic and perischemic areas of cortex and striatum at Bregma level. All quantitative analyses described here below were performed blind to the treatment by two independent experimenters and data were averaged and expressed as mean ± SEM of number cells per optical field of “*n*” animals.

#### 2.3.7. Determination of TNF-α and IL-10 in the plasma

The level of Tumour Necrosis Factor-α (TNF-α) pro-inflammatory cytokine and of interleukine-10 (IL-10) a regulatory cytokine, were measured on aliquots (100 µl) of plasma from sham-operated rats (*n* = 3), vehicle-treated rats (*n* = 3), ACTZ-treated rats (*n* = 3) and AN11-740-treated (*n* = 3) rats, using commercial ELISA kits (Rat TNF-α Platinum ELISA, Catalogue no: BMS622, Affymetrix eBioscience, Vienna, Austria; Rat IL-10 Platinum ELISA, Catalogue no: BMS629, Affymetrix eBioscience, Vienna, Austria), following the protocol provided by the manufacturer. Results are expressed as pg of protein/ml of plasma.

#### 2.3.8. Statistical analysis

Data were statistically analysed by one-way analysis of variance (ANOVA) followed by Newman-Keuls multiple comparison test and by unpaired Student's *t* test, as appropriate. A value of *p* < 0.05 was considered statistically significant. The statistical analysis was performed utilising GraphPad Prism7.

## 3. Results

### 3.1. The application of selective CAIs significantly delayed the appearance of AD induced by 30 min OGD

**Figure 2.**
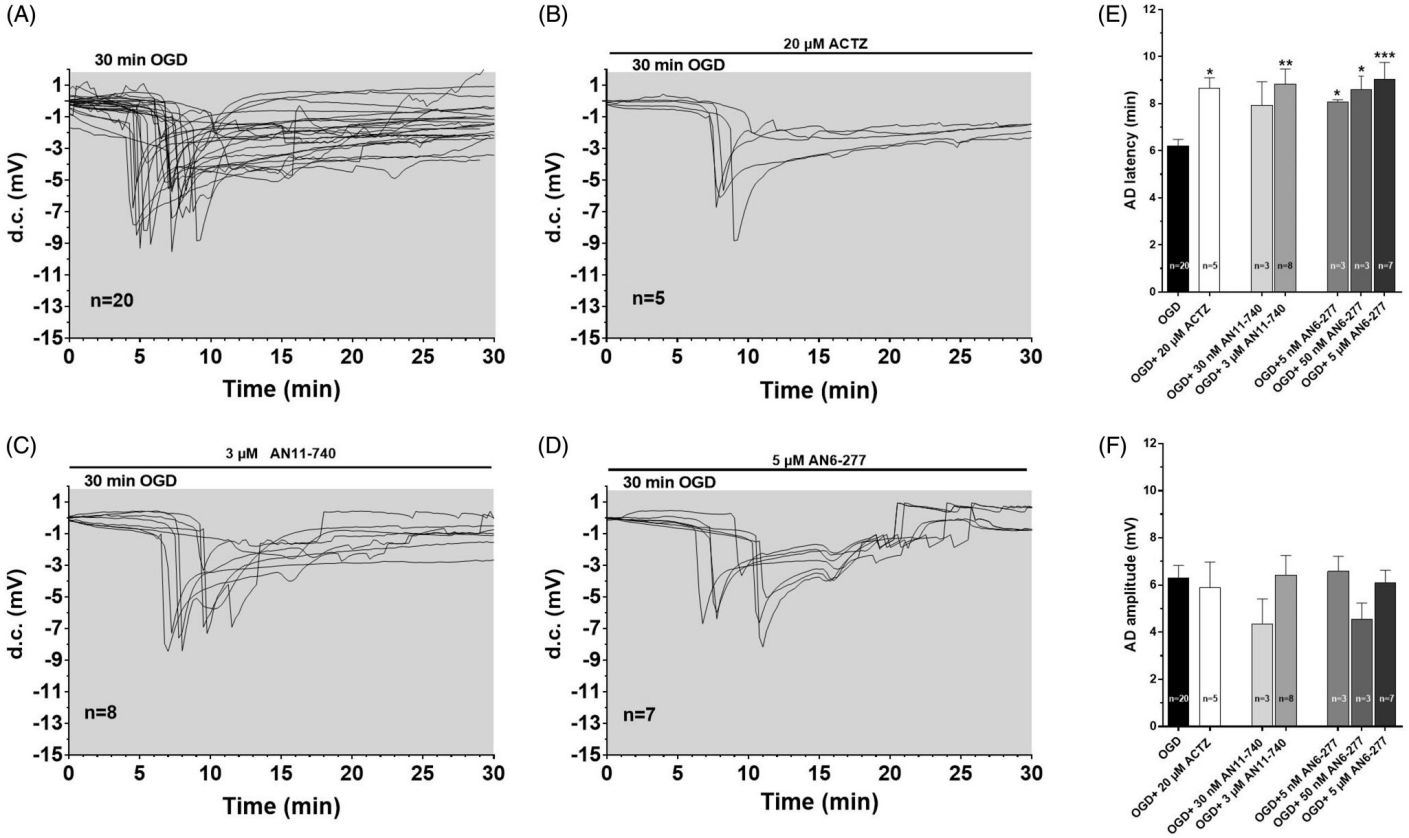
Effects of different CA inhibitors on AD development during 30 min OGD in the CA1 region. (A–D) The graphs show the d.c. shift traces during 30 min OGD in untreated OGD slices (A, *n* = 20), in the presence of 20 µM ACTZ (B, *n* = 5), 3 µM AN11-740 (C, *n* = 8) and 5 µM AN6-277 (D, *n* = 7). Each inhibitor was applied at least 20 min before OGD and maintained for all the insult. (E) Each column represents the mean ± SEM of AD latency recorded in hippocampal slices during 30 min OGD in different experimental groups. AD was measured from the beginning of OGD insult. *p < 0.05, **p < 0.01, ***p < 0.001 vs. OGD, One-way ANOVA followed by Bonferroni *post hoc* test. (F): Each column represents the mean ± SEM of AD amplitude recorded in the CA1 region during 30 min OGD. The number of slices is reported in the columns.

The experiments were performed on 49 slices taken from 20 rats to test the role of CAIs during severe, 30 min long OGD, a time-duration that is invariably harmful for the tissue^30^^,^^38^. The effects of two new selective CAIs, AN6-277 and AN11-740, on the time of the AD appearance and amplitude, were evaluated (Figure 2) and compared to those obtained in the presence of the prototypical CAI, ACTZ. In accord with our previous data^30^^,^^39^ no recovery of fEPSP was recorded after interruption of 30 min OGD in all experimental conditions (not shown). As illustrated in Figure 2(A), 30-min OGD elicited the appearance of AD in untreated OGD slices, which presented a mean latency of 6.2 ± 0.3 min (calculated from the beginning of OGD; *n* = 20, Figure 2(A,E)) and a mean peak amplitude of −6.3 ± 0.5 mV (*n* = 20, Figure 2(A,F)). When OGD was applied in the presence of CAIs, the d.c. shifts were always delayed (Figure 2(B–E)). Indeed, the latency of AD was postponed to 8.65 ± 0.5 min in the presence of 20 µM ACTZ, (*n* = 5, *p<*0.05, Figure 2(B,E)), to 8.8 ± 0.6 min in the presence of 3 µM AN11-740 (*n* = 8, *p<*0.01, Figure 2(C,E)) and to 9.04 ± 0.7 min in the presence of 5 µM AN6-277 (*n* = 7, *p<*0.001, Figure 2(D,E)). AN6-277 affected AD latency also when tested at lower concentrations (Figure 2(E)). All compounds tested did not significantly change AD amplitude in comparison to that found in OGD untreated slices (Figure 2(F)).

### 3.2. Effect of treatment with CAIs on neurological deficit after pMCAo

**Figure 3.**
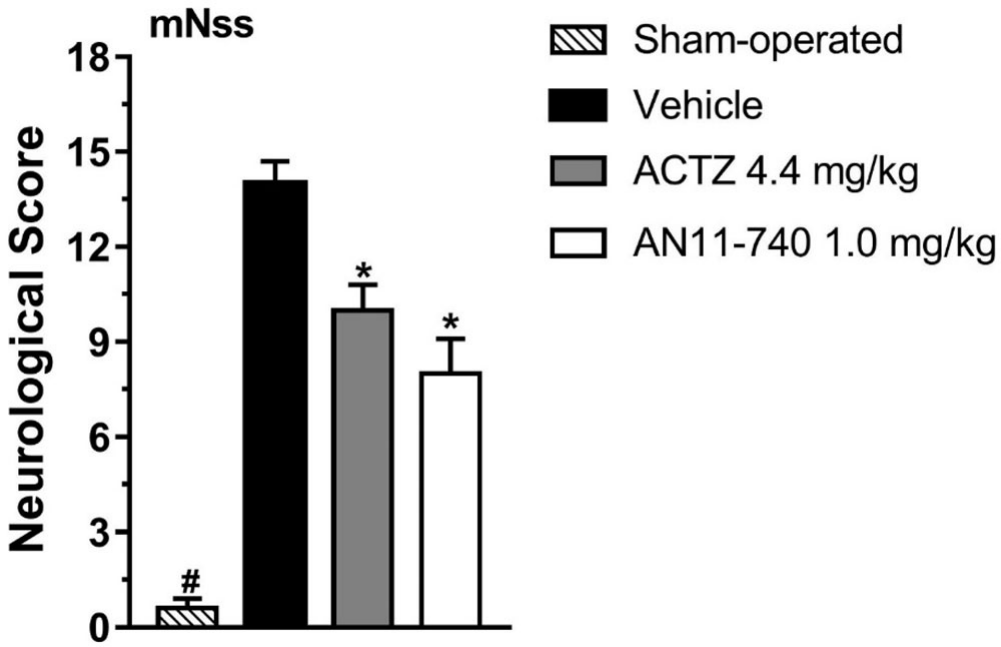
Effect of sub-chronic treatment with ACTZ and AN11-740 on neurological deficit, 24 h after pMCAo. mNSS test: the score is evaluated 24 h after pMCAo in sham-operated (*n* = 7), vehicle-treated (*n* = 4), ACTZ-treated (*n* = 4) and AN11-740-treated (*n* = 5) rats. Data are expressed as mean ± SEM, one-way ANOVA followed by Newman-Keuls *post hoc* test: ^#^*p* < 0.001 sham-operated rats vs. vehicle- ACTZ- AN11-740-treated rats; *at least *p* < 0.01 ACTZ- and AN11-740-treated rats vs. vehicle-treated rats.

The mNSS test, performed according to^40^, shown in Figure 3, indicated that sham-operated rats had a neurological score of 0.6 ± 0.3 (mean ± SEM), 24 h after pMCAo. Vehicle-treated rats showed a clear neurological deficit with a neurological score of 14.0 ± 0.7 that defines a severe injury. Sub-chronic treatment with both CAIs, ACTZ (4.4 mg/kg i.p.) and AN11-740 (1.0 mg/kg i.p.) significantly reduced the neurological score by 28.6 and by 42.9%, respectively, 24 h after pMCAo (One-way ANOVA: F_3,16_ = 67.24, *p* < 0.0001; Newman-Keuls *post hoc* test: **at least *p* < 0.01). Sham-operated rats had a neurological score significantly different from vehicle-, ACTZ- and AN11-740-treated rats (One-way ANOVA: F_3,16_ = 67.24, *p* < 0.0001; Newman-Keuls *post hoc* test: **at least *p* < 0.01; ***p < 0.001).

### 3.3. Effect of treatment with CAIs on brain ischaemic damage after pMCAo

**Figure 4.**
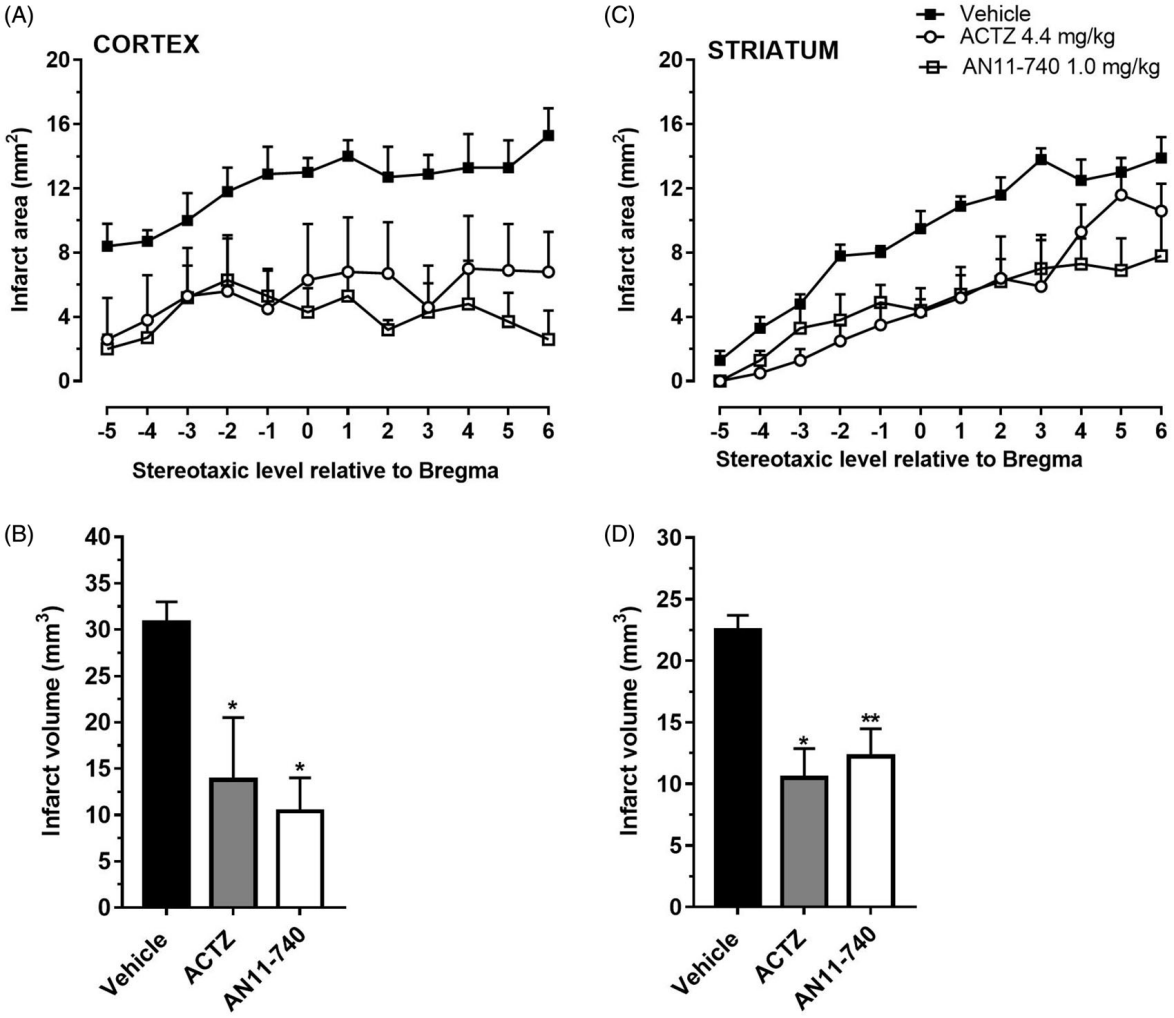
Effect of sub-chronic treatment with ACTZ and AN11-740 on infarct area (A,C) and infarct volume (B,D) in the cortex and striatum 24 h after pMCAo. (A,C) Data represent infarct area measured at 12 predetermined coronal levels through the brain of vehicle-treated (*n* = 4), ACTZ-treated (*n* = 4) and AN11-740-treated (*n* = 5) rats. Bregma = 0 [37]. (B,D) Bar graphs indicate the infarct volume calculated as mean ± SEM in the striatum and cortex. One-way ANOVA followed by Newman–Keuls *post hoc* test: *p < 0.05 and **p < 0.01 vs. vehicle-treated rats.

**Figure 5.**
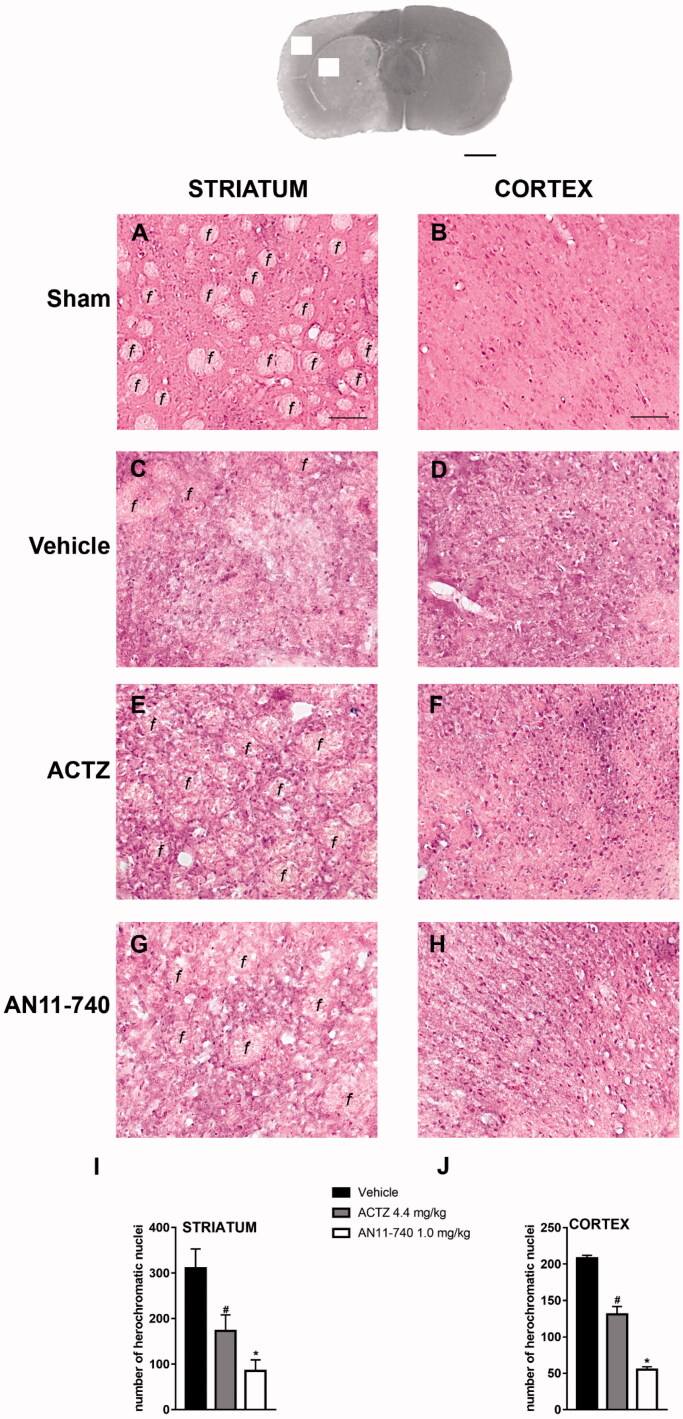
Effect of sub-chronic treatment with ACTZ and AN11-740 on the cytoarchitecture of the striatum and cortex 24 h after pMCAo. Upper part: Representative photomicrograph of a coronal section (at Bregma = 0^36^,) showing the ischaemic area in a vehicle-treated rat. The two white squares indicate regions within the ischaemic area where photomicrographs were captured. Scale bar = 2 mm. (A–H) Representative microphotographs of H&E staining from dorsal striatum and fronto-parietal cortex of a sham-operated (A,B), a vehicle- (C,D), an ACTZ- (E,F) and an AN11-740-treated rat (G, H). The white matter *fascicula* (*f*) are evidenced. Scale bar = 100 µm. (I,J) Quantitative analyses of heterochromatic nuclei per striatal (I) and cortical areas (F) at coronal level AP = 0 from Bregma. Data represent the mean ± SEM of 3 rats/group. One-way ANOVA followed by Newman-Keuls *post hoc* test: ^#^at least *p* < 0.05 ACTZ- vs. vehicle-treated rats; *at least *p* < 0.001 AN11-740- vs. vehicle-treated rats.

Figure 4 shows the extent of ischaemic damage evaluated as infarct area (Figure 4(A,C)) and infarct volume (Figure 4(B,D)) in ischaemic striatum and cortex of vehicle-, ACTZ and AN11-740-treated rats, 24 h after pMCAo. Sub-chronic treatment with both ACTZ and AN11-740 significantly reduced the infarct volume in ischaemic cortex by 55.2 and 66.2%, respectively (One-way ANOVA: F_2,10_ = 5.9, *p* < 0.02; Newman-Keuls *post hoc* test: *p < 0.05) and in ischaemic striatum by 53.4 and 45.6%, respectively (One-way ANOVA: F_2,9_ = 9.5, *p* < 0.006; Newman-Keuls *post hoc* test: *at least *p* < 0.05). Sham-operated rats did not show any damage. To characterise the cytoarchitecture of the ischaemic cortex and ischaemic striatum 24 h after pMCAo, ischaemic tissue was stained by H&E (Figure 5(A–H)). Twenty-four hours after ischaemia, the typical cytoarchitecture of these two regions (for a description see [41]) was lost. In sham-operated rats, the typical caudate-putamen cytoarchitecture of the dorsal striatum was appreciable (Figure 5(A)), with numerous transversally sectioned white matter fascicula (*f*) surrounded by grey matter containing diverse types of neurons, distinct on the bases of their size and shape. In the fronto-parietal cortex (Figure 5(B)), the typical columnar organisation was recognisable^41^. In vehicle-treated rats, the striatal tissue was clearly damaged, the cytoarchitecture was lost, the *fascicula* were much less recognisable, the distinction between white and grey matter no more appreciable, the interstitial spaces increased, and numerous heterochromatic small nuclei were present (Figure 5(C)). In the fronto-parietal cortex, the columnar organisation was hardly visible, the interstitial spaces were enlarged and dilated, and numerous heterochromatic small nuclei were present (Figure 5(D)). Qualitative analysis (Figure 5(E–H)) shows that administration of both ACTZ and AN11-740 was associated with a recovery of tissue cytoarchitecture, and with a reduction of heterochromatic small nuclei in both brain regions. The white matter *fascicula* were recognisable in the dorsal corpus striatum (Figure 5(E,G)), and the columnar organisation was appreciable in the fronto-parietal cortex (Figures 5(F,H)). Quantitative analysis showed that treatment with both ACTZ and AN11-740 significantly reduced the number of heterochromatic nuclei in the ischaemic striatum (One-way ANOVA: F_2,6_ = 10.1, *p* < 0.001; Newman-Keuls *post hoc* test: *at least *p* < 0.05, Figure 5(I)) and in the ischaemic cortex (One-way ANOVA: F_2,6_ = 115.6, *p* < 0.0001; Newman-Keuls *post hoc* test: ***at least *p* < 0.001, Figure 5(J)).

### 3.4. Effect of treatment with CAIs on neuronal damage after pMCAo

**Figure 6.**
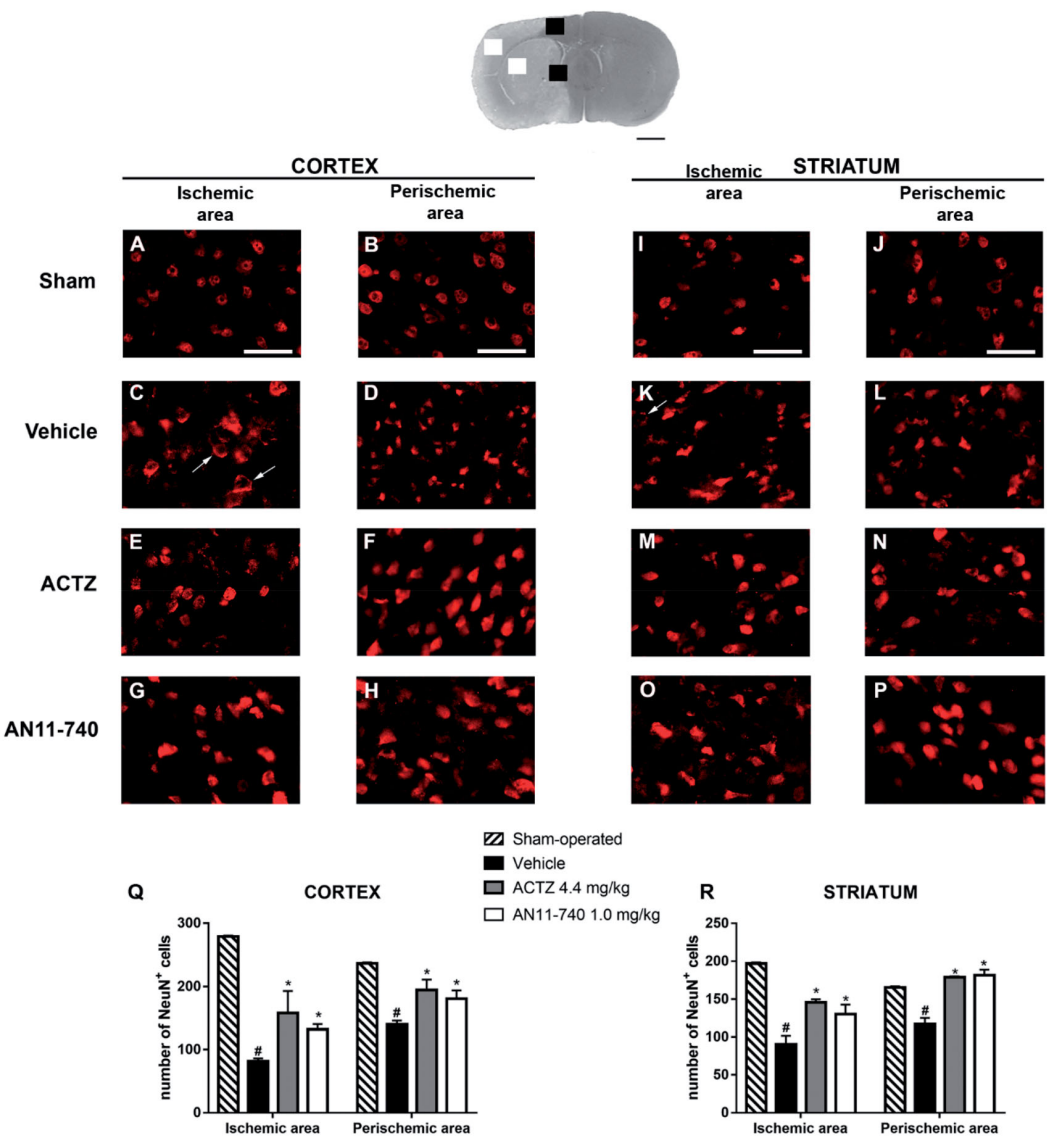
Effect of sub-chronic treatment with ACTZ and AN11-740 on neuronal damage in cortex and striatum 24 h after pMCAo. Upper part: Representative photomicrograph of a coronal section (at Bregma = 0,^37^) showing the ischaemic area in a vehicle-treated rat. The two white and black squares indicate regions within the ischaemic area and within the perischemic area, respectively, where photomicrographs were captured. Scale bar = 2 mm. (A–P) Representative microphotographs of neurons (red) in cortical ischaemic and perischemic areas and in striatal ischaemic and perischemic areas of sham-operated (A, B; I, J; *n* = 3), vehicle- (C, D; K, L; *n* = 4) ACTZ- (E, F; M, N; *n* = 4) and AN11-740- (G, H; O, P; *n* = 5) treated rats. Scale bar = 50 µm. (Q, R) Quantitative analysis of NeuN^+^ cells in cortical and striatal ischaemic and perischemic areas, bar graphs represent mean ± SEM of the number of neurons per optical field (20×). One-way ANOVA followed by Newman–Keuls *post hoc* test: ^#^at least *p* < 0.01 vehicle-treated vs. sham-operated rats; *at least *p* < 0.05 ACTZ- and AN11-740- vs. vehicle-treated rats.

Twenty-four hours after pMCAo, the extent of neuronal damage in cortical and striatal ischaemic areas and perischemic areas was assessed by immunohistochemistry using anti-NeuN antibody. Representative images of NeuN immunostaining of each rat group are shown in Figure 6(A–P). In the ischaemic cortex and striatum of vehicle-treated rats, we observed the presence of many damaged neurons characterised by shrunken cell bodies^42^ (Figure 6(C,D,K,L)) or by damaged nuclei that had lost NeuN^+^ immufluorescence, while the immunofluorescence persisted in the cytoplasm (shown by the arrows in Figures 6(C,K)). Sub-chronic treatment with CAIs antagonised neuronal damage in both ischaemic area (Figure 6(E,G; M,O)) and perischemic area (Figure 6(F, H; N, P)). Figures 6(Q,R) show the quantitative analysis of NeuN^+^ cells in the ischaemic and in the perischemic areas of both cortex and striatum of sham-operated, vehicle- ACTZ- and AN11-70-treated rats. Statistical analysis performed by One-way ANOVA evidenced that the number of NeuN^+^ cells significantly decreased both in the cortical ischaemic and perischemic areas (One-way ANOVA: F_3,11_ = 28.9, *p* < 0.0001; Newman-Keuls *post hoc* test: ***p < 0.001; One-way ANOVA: F_3,12_ = 9.3, *p* < 0.001; Newman-Keuls *post hoc* test: **p < 0.01; Figures 5(Q,R)) and in the striatal ischaemic and perischemic areas (One-way ANOVA: F_3,11_ = 15.4, *p* < 0.0003; Newman–Keuls *post hoc* test: ***p < 0.001; One-way ANOVA: F_3,12_ = 22.96, *p* < 0.0001; Newman–Keuls *post hoc* test: ***p < 0.001; Figures 5(Q,R)) of vehicle-treated rats in comparison to sham-operated rats. Sub-chronic treatment with both ACTZ and AN11-740 significantly counteracted the decrease in neuron number in ischaemic areas (One-way ANOVA followed by Newman-Keuls *post hoc* test: *p < 0.05; Figures 6(Q,R)) and in perischemic areas (One-way ANOVA followed by Newman-Keuls *post hoc* test: *at least *p* < 0.05; Figures 6(Q,R)) of both cortex and striatum.

### 3.5. Effect of treatment with CAIs on microglia activation after pMCAo

**Figure 7.**
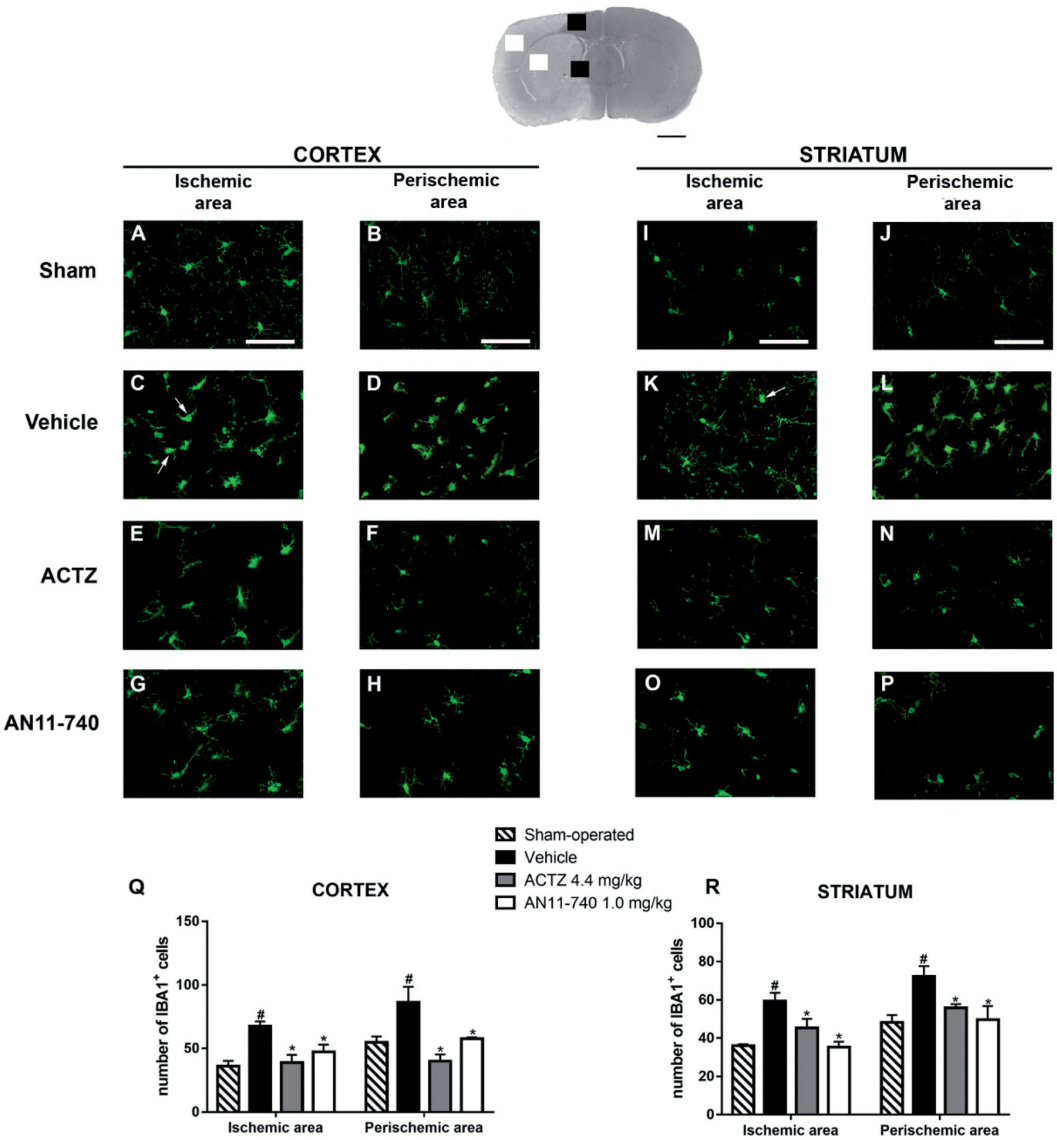
Effect of sub-chronic treatment with ACTZ and AN11-740 on microglia activation in cortex and striatum 24 h after pMCAo. Upper part: Representative photomicrograph of a coronal section (at Bregma = 0^36^) showing the ischaemic area in a vehicle-treated rat. The two white and black squares indicate regions within the ischaemic area and within the perischemic area, respectively, where photomicrographs were captured. Scale bar = 2 mm. (A–P) Representative microphotographs of microglia (green) in cortical ischaemic and perischemic areas and in striatal ischaemic and perischemic areas of sham-operated (A, B; I, J, *n* = 3), vehicle- (C, D; K, L, *n* = 3) ACTZ- (E, F; M, N, *n* = 4) and AN11-740- (G, H; O, P, *n* = 3) treated rats. Scale bar = 50 µm. (Q, R) Quantitative analysis of IBA1^+^ cells in cortical and striatal ischaemic and perischemic areas, bar graphs represent mean ± SEM of the number of microglia cells per optical field (20×). One-way ANOVA followed by Newman–Keuls *post hoc* test: ^#^at least *p* < 0.05 vehicle-treated vs. sham-operated rats; *at least *p* < 0.05 ACTZ- and AN11-740- vs. vehicle-treated rats.

Twenty-four hours after ischaemia, microglia cells were characterised using immunofluorescent staining with anti-IBA1 antibody, as shown by the representative images of Figure 7(A–P). In sham-operated rats, resting microglia was diffusely distributed throughout the cortex and the striatum and was characterised by small cell body and thin and ramified branches (Figure 7(A,B; I,J)). Twenty-four hours after pMCAo, we found a strong pattern of activation of microglia cells, both in the ischaemic cortex and ischaemic striatum of vehicle-treated rats. In the cortical and striatal ischaemic areas, microglia cells appeared round-shaped and amoeboid, morphology typical of activated cells (shown by the arrows in Figure 7(C,K)). In the cortical and striatal perischemic areas, microglia showed a hyperthrophic cell body with thick and short processes, a morphology defined reactive microglia^43^^,^^44^ (Figure 7(D,L)). Sub-chronic treatment with both ACTZ and AN11-740 appeared to revert the morphological alterations caused by ischaemia in both ischaemic (Figure 7(E,G; M,O)) and perischemic areas (Figure 7(F,H; N,P)). Quantitative analysis perfomed by One-way ANOVA showed that IBA1^+^ cells increased in vehicle-treated rats both in cortical ischaemic and perischemic areas (One-way ANOVA: F_3,8_ = 7.9, *p* < 0.008; Newman–Keuls *post hoc* test: **p < 0.01; One-way ANOVA: F_3,8_ = 7.4, *p* < 0.01; Newman-Keuls *post hoc* test: *p < 0.05; Figure 7(Q)) and both in striatal ischaemic and perischemic areas (One-way ANOVA: F_3,9_ = 8.0, *p* < 0.006; Newman-Keuls *post hoc* test: **p < 0.01; One-way ANOVA: F_3,9_ = 5.4, *p* < 0.02; Newman–Keuls *post hoc* test: *p < 0.05; Figure 7(R)) in comparison to sham-operated rats. Sub-chronic treatment with both CAIs significantly reduced the number of IBA1^+^ cells in ischaemic (One-way ANOVA followed by Newman–Keuls *post-hoc* test: *at least *p* < 0.05; Figure 7(Q,R)) and perischemic areas (One-way ANOVA followed by Newman–Keuls *post-hoc* test: *at least *p* < 0.05; Figure 7(Q,R)) of both ischaemic cortex and striatum.

### 3.6. Effect of treatment with CAIs on astrocytes alteration after pMCAo

**Figure 8.**
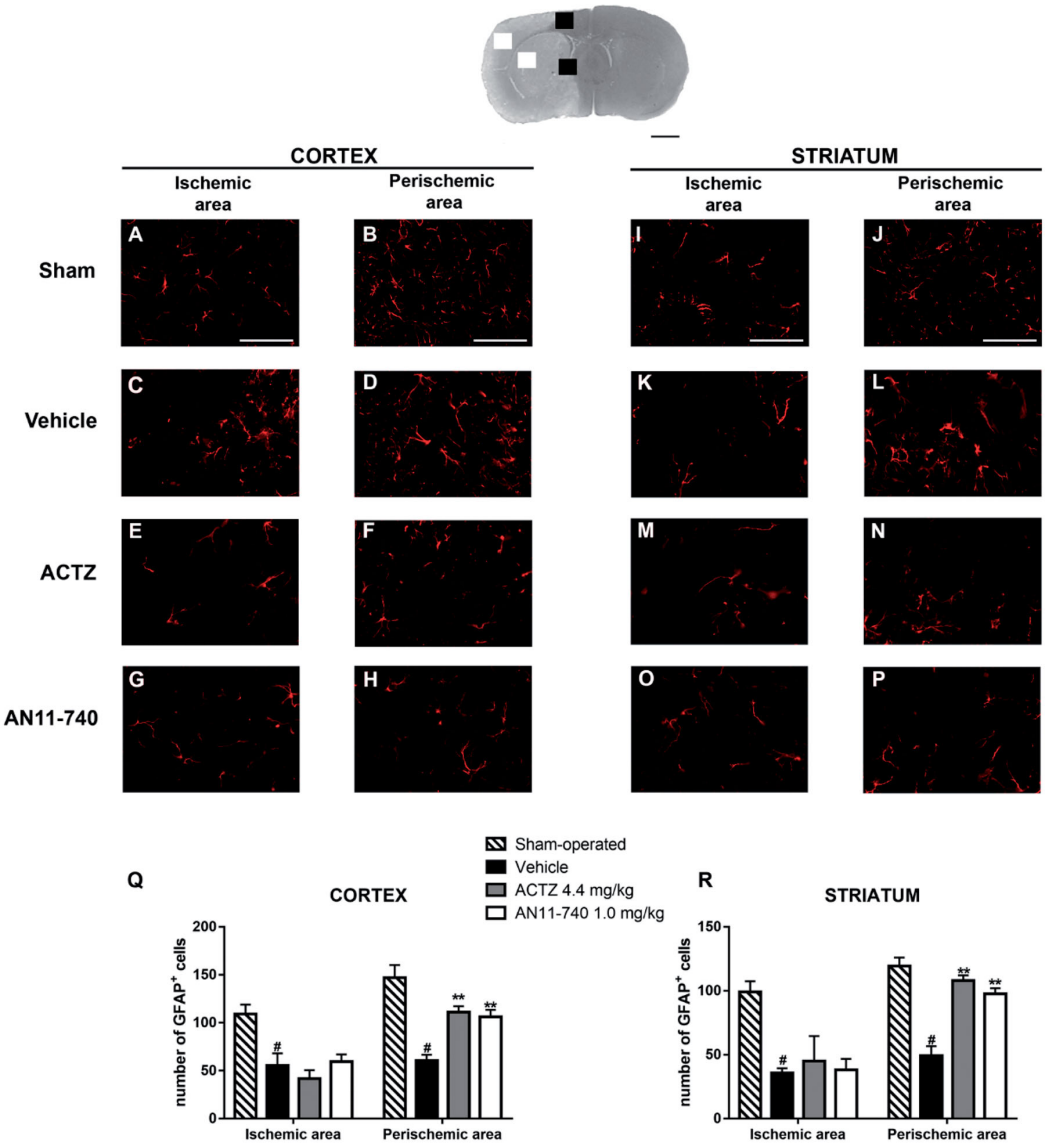
Effect of sub-chronic treatment with ACTZ and AN11-740 on astrocytes alteration in cortex and striatum 24 h after pMCAo. Upper part: Representative photomicrograph of a coronal section (at Bregma = 0^36^) showing the ischaemic area in a vehicle-treated rat. The two white and black squares indicate regions within the ischaemic area and within the perischemic area, respectively, where photomicrographs were captured. Scale bar = 2 mm. (A–P) representative microphotographs of astrocytes (red) in cortical ischaemic and perischemic areas and in striatal ischaemic and perischemic areas of sham-operated (A, B; I, J, *n* = 3), vehicle- (C, D; K, L, *n* = 3) ACTZ- (E, F; M, N, *n* = 4) and AN11-740- (G, H; O, P, *n* = 3) treated rats. Scale bar = 50 µm. (Q, R) Quantitative analysis of GFAP^+^ cells in cortical and striatal ischaemic and perischemic areas, bar graphs represent mean ± SEM of the number of astrocytes per optical field (20×). One-way ANOVA followed by Newman–Keuls *post hoc* test: ^#^at least *p* < 0.05 vehicle-treated vs. sham-operated rats; **at least *p* < 0.01 ACTZ- and AN11-740- vs. vehicle-treated rats.

Immunofluorence analysis using anti-GFAP antibody specific for astrocytes, are shown by the representative images of Figure 8(A–P). In sham-operated rats, resting astrocytes were diffusely distributed throughout the cortex and the striatum, and presented small cell body and faintly stained thin processes (Figure 8(A,B; I, J)). Twenty-four hours after ischaemia, in cortical and striatal ischaemic areas of vehicle-treated rats, GFAP^+^ cells showed small cell bodies and fragmented branches (Figure 8 (C,K)), morphological features typical of suffering cells. In cortical and striatal perischemic areas of vehicle-treated rats, astrocytes have hyperthrophic cell bodies and long and thick processes (Figure 8 (D,L)), characteristic of reactive cells^45^. Sub-chronic treatment with ACTZ and AN11-740 amiliorates astrocytes morphology both in ischaemic cortex and ischaemic striatum (Figure 8(E–H; M–-P)): astrocytes appeared less hyperthrophic and less activated. Quantitative analysis evidenced that the number of GFAP^+^ cells decreased in vehicle-treated rats both in cortical ischaemic and perischemic areas (One-way ANOVA: F_3,8_ = 8.7, *p* < 0.006; Newman–Keuls *post hoc* test: *p < 0.05; One-way ANOVA: F_3,8_ = 16.4, *p* < 0.0009; Newman-Keuls *post hoc* test: ***p < 0.001; Figure 8(Q)) and both in striatal ischaemic and perischemic areas (One-way ANOVA: F_3,8_ = 6.6, *p* < 0.01; Newman–Keuls *post hoc* test: *p < 0.05; One-way ANOVA: F_3,9_ = 28.3, *p* < 0.0001; Newman–Keuls *post hoc* test: ***p < 0.001; Figure 8(R)) in comparison to sham-operated rats. Sub-chronic treatment with CAIs didn’t modify the number of GFAP^+^ cells neither in the cortical nor in the striatal ischaemic areas (Figure 8(Q,R)), but partially conteracted the decrease in number of astrocytes in the cortical and striatal perischemic areas (One-way ANOVA followed by Newman–Keuls *post-hoc* test: **p < 0.01; Figure 8(Q,R); One-way ANOVA followed by Newman–Keuls *post hoc* test: ***at least *p* < 0.001; Figure 8(Q,R)).

### 3.7. Effect of treatment with CAIs on cytokine plasma levels after pMCAo

**Figure 9.**
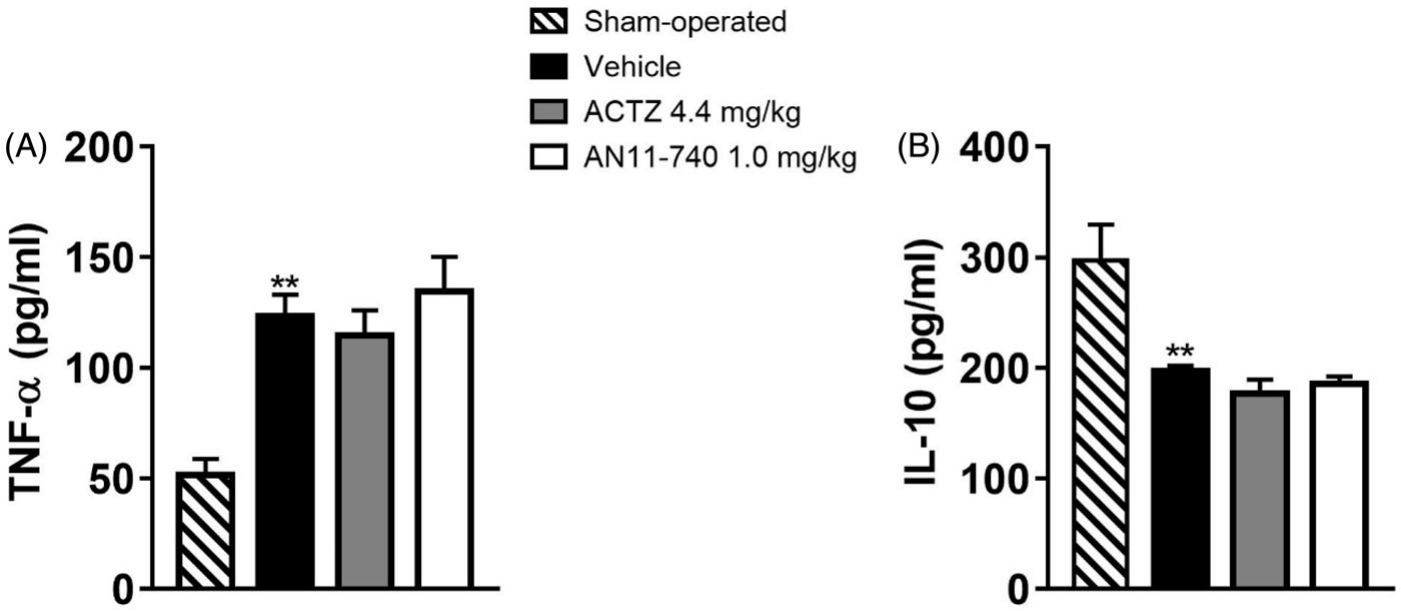
Effect of sub-chronic treatment with ACTZ and AN11-740 on TNF-α (A) and IL-10 (B) plasma levels. Results are expressed as pg of protein/ml of plasma and values are mean ± SEM. One-way ANOVA followed by Newman–Keuls *post hoc* test: **p < 0.01 vs. sham-operated rats.

Twenty-four hours after pMCAo, we evaluated plasma levels of the pro-inflammatory cytokine TNF-α, and of IL-10, a regulatory cytokine with an anti-inflammatory action. In vehicle-treated rats TNF-α plasma levels significantly increased as compared to sham-operated rats (One-way ANOVA: F_3,8_ = 10.7, *p* < 0.003; Newman–Keuls *post hoc* test: **p < 0.01; Figure 9(A)) while the plasma level of IL-10 was significantly reduced in vehicle-treated rats as compared to sham-operated rats (One-way ANOVA: F_3,7_ = 13.7, *p* < 0.002; Newman–Keuls *post hoc* test: **p < 0.01; Figure 9(B)). Sub-chronic treatment with both CAIs didn’t modify neither TNF-α nor IL-10 plasma levels 24 h after pMCAo (Figure 9(A,B)).

## 4. Discussion and conclusions

In the present work we report that ACTZ and two different sulphonamide CAIs, AN11-740 and AN6-277, protect from a strong neuron depolarisation induced in a “similary-ischemic” model in hippocampal slices. Furthermore, ACTZ and the highly lipophilic compound (AN11-740), systemically and sub-chronically administered in the rat, revealed highly protective also from *in vivo* ischaemia i.e. against neurological deficit and against the ischaemic damage to all neural cells induced by pMCAo. In agreement with previous results^30^^,^^46^^,^^47^, we report that a severe OGD, 30 min long, induced the appearance of the electrophysiological phenomenon of AD recorded in all the hippocampal slices. AD is an early and critical event after ischaemia, demonstrated both *in vivo*^48^ and *in vitro*^30^^,^^49^, and consists in a robust neuronal depolarisation seen as a negative d.c. shift of membrane potential. AD triggers a broad range of molecular events which lead to cell death and represents an unequivocal sign of neuronal injury^48^, as indicated by the fact that after its appearance, the evoked field potential was permanently lost^30^^,^^46^. Recurring peri-infarct depolarisation arises at the border of the ischaemic *core* during the first 3–4 h poststroke^50–54^. It is well established that sustained activation of N-methyl-d-aspartate (NMDA)-type glutamate receptors is essential to AD initiation and propagation leading to excitotoxic neuronal death in stroke^48^. Therefore, it is well accepted that a pharmacological treatment that postpones the onset of a perinfarct depolarisation helps to protect brain tissue after ischaemia^48^^,^^55^.

The maintenance of pH homeostasis in the CNS is pivotal for neurotransmission mechanisms and variations from this homeostasis are crucial for processes underlying a spectrum of pathological conditions including ischaemia^16^. Indeed, under ischaemia, the loss of oxygen caused by hypoxia leads to a switch from aerobic to anaerobic glucose metabolism, and subsequently increased production of lactic acid and lowered intracellular pH^14^^,^^56^ in neurons and glial cells^16^.

Neurons are particularly sensitive to the pH decrease^16^ and acidosis augments the vulnerability of glia to injury induced by OGD^57^. Indeed, changes in the intracellular pH may affect neurotransmitters release. Lowering of pH results in increased release of dopamine^58^^,^^59^, noradrenaline and serotonin from rat brain synaptosomes^59^. Glial acidosis has been shown to trigger glial glutamate release and neuronal cell death^60^.

The CA isoforms IV, IX and XII, all extracellular and membrane-bound enzymes, are highly expressed in glial cells^61^^,^^62^ and contribute to pH homeostasis, both in physiological and pathological conditions. Astrocytes have a key role in pH regulation in the brain^63^. Glial CA converts neuron derived CO_2_ to bicarbonate and protons which are extruded of the glial cell by a Na^+^/HCO_3_^-^ cotransporter and monocarboxylate transporters^64^. Extracellularly CA is pivotal in buffering extracellular pH by recycling CO_2_ in bicarbonate and protons^61^. The evidence that under hypoxic conditions, the two CA isoforms IX and XII increase^17–19^ supports the possibility that during OGD, the sulphonamides ACTZ, AN6-277, and AN11-740, by reducing the activity of CA isoforms, reduce the concentration of hydrogen ions, the excitatory amino acid efflux and therefore the participation of glutamate in triggering the AD.

Twenty-four hours after *in vivo* ischaemia induced by pMCAo, a clear infarct volume was detected and the immunohistochemical analysis revealed a definite ischaemic damage both in ischaemic and perischemic areas of striatum and cortex and a clear decrease of neuron number. ACTZ and AN11-740 have definitely protected neurons from damage and from the reduction in number both in the cortex and in striatum. In an earlier study, CAIs of the sulphonamide or coumarin type used at different doses and administration route were shown to improve neurological deficit, but not to significantly influence the ischaemic damage 24 h after pMCAo^65^.

H&E staining shows that sub-chronic treatment with CAIs re-established the cytoarchitecture in cortex and striatum and decreased heterochromatic small nuclei that belong to astrocytes and microglia^66^. Twenty-four hours after pMCAo, a pattern of strong microglia activation and proliferation was found. Astrocytes had morphological features typical of suffering cells and were reduced in number in ischaemic and perischemic areas of both cortex and striatum. Sub-chronic treatment with CAIs reduced microglial activation and proliferation and ameliorated astrocyte suffering pattern and degeneration in perischemic areas of the cortex and striatum. In cortical and striatal ischaemic areas, few GFAP^+^ cells were detectable in agreement with previous observations and^45^^,^^67^ although CAIs appear to ameliorate astrocyte suffering features, they did not protect from astrocyte degeneration. CAIs by re-establishing H^+^ concentration and thus contributing to the pH homeostasis during ischaemia^13–15^ can protect from ischaemic injury because of reducing excitatory amino acid outflow and ensuing excitotoxicity. Evidence that CAIs are protective *in vitro* from an acute OGD-induced depolarisation support the fact that CAIs protect *in vivo* from the functional and tissue damage because of a direct central effect in decreasing excitotoxicity and precocious brain depolarisation.

Twenty-four hours after pMCAo we found an increase of TNF-α in the peripheral plasma, in agreement with previous results^68^. Moreover, we found a decrease of IL-10, a regulatory cytokine with an anti-inflammatory action. A TNF-α increase and an IL-10 decrease in the systemic circulation represent valuable markers over time of an ischaemic “secondary damage” due to the production of inflammation mediators following an ischaemic event^69^. Treatment with ACTZ and AN11-740 did neither modified TNF-α or IL-10 levels in the plasma. However, a 24 h after stroke time lapse is too early after the damage to appreciate a protective effect of the CAIs from a later ischaemic “secondary damage”. In conclusion, our data indicate that an early treatment with highly liposoluble sulphonamide CAIs could be a useful treatment to complement the thrombolytic application in the therapeutic time-window after cerebral ischaemia.
